# Antibiotic Treatment of Dogs and Cats during Pregnancy

**DOI:** 10.4061/2010/385640

**Published:** 2010-12-14

**Authors:** Marcela Rebuelto, María Elena Loza

**Affiliations:** ^1^Farmacología, Facultad de Ciencias Veterinarias, Universidad de Buenos Aires, Chorroarín 280, 1427 Buenos Aires, Argentina; ^2^Teriogenología, Facultad de Ciencias Veterinarias, Universidad de Buenos Aires, Chorroarín 280, 1427 Buenos Aires, Argentina

## Abstract

The use of pharmacological agents in pregnant females poses a major clinical challenge due to the marked physiological changes that may modify the pharmacokinetics of drugs and to the potential effects on the fetus. The purpose of this paper is to review briefly our knowledge on the use of antibacterial drugs during pregnancy and to provide information for the judicious selection of an antimicrobial treatment for use in pregnant bitches and queens. The risk to the fetus is a result of the ability of a drug to reach the fetal circulation and to produce toxic effects. The placenta functions as a barrier that protects the fetus due to the presence of transporters and metabolising enzymes; however, during pregnancy, the presence and activity of both enzymes and transporters may change. Antimicrobial agents that have been shown to be safe for use during pregnancy include betalactams, macrolides, and lincosamides. Pharmacotherapy during pregnancy in all species may affect adversely the developing fetus; therefore, it should be avoided when possible.

## 1. Introduction

The use of pharmacological agents in pregnant females poses a major clinical challenge. On the one hand, the marked physiological changes caused by the pregnancy may produce modifications in the pharmacokinetics (absorption, distribution, biotransformation, and excretion) of drugs which may require adjustment of dosage. On the other hand, the transplacental transfer of drugs from the maternal to the fetal blood and tissues, leading to potential effects on the fetus, is a major concern. Thus, both mother and fetus must be included in the risk/benefit assessment to ensure a rational decision, weighing the therapeutic benefits of the treatment to the mother against its potential harm to the fetus [1–5]. 

Currently, there is little information on pharmacological treatments during pregnancy in veterinary medicine. Several factors may account for this situation. First, there are valid ethical concerns in conducting research on pregnant females, even in nonhuman species as our domestic animals [6]. Second, even though a variety of animal models has been used to investigate various aspects of pregnancy, there are marked differences between species, making unwise the extrapolation of data obtained from one species to another [7–11]. Third, changes during pregnancy do not occur in one step, but are dynamic, and they are establishing marked differences between individuals of the same species and even in the same individual between different stages of the pregnancy [12–19]. Fourth, even though some *ex vivo/in vitro* methods for studying transplacental transfer have proved useful, such as the dual recirculating placental perfusion model and the use of transporters overexpressing cell lines, they still have the limitations of experiences carried out in isolated organs or cell cultures [20–22]. Fifth, the current conservative approach, not only in human but also in veterinary medicine, is to avoid medical treatments in pregnant females as much as possible; therefore, data from clinical observations are sparse. 

The purpose of this paper is to review briefly our knowledge on the use of drugs during pregnancy, as antibacterial therapy is the most likely pharmacotherapy to be administered to a pregnant bitch or queen, and to provide information for the judicious selection of an antimicrobial treatment for use during pregnancy.

## 2. Pharmacokinetics in Pregnancy

Pharmacological effects of drugs are related to the drug's concentration at the site of action. Low (subtherapeutical) levels may lead to therapeutic failures; on the contrary, high (supratherapeutical) levels may produce toxic effects. Changes in any of the physiological processes occurring after the administration of a drug are the reasons of the concerns in the pharmacological management of medical conditions in pregnant females [23–25]. Therapeutic dosage regimens of drugs, that is, dose and dosage interval, are generally calculated according to major pharmacokinetic parameters, primarily total body clearance and volume of distribution, obtained from studies conducted in healthy non pregnant individuals. The physiological changes during pregnancy may modify the absorption, distribution, and rate of elimination of a drug to an extent that dose adjustment would be required for its safe and clinically effective use. 

The high progesterone concentration during pregnancy induces a reduction in the gastrointestinal motility and increases the intestinal blood flow. These modifications may affect (increase, decrease, or neither) the oral absorption of a drug and hence its bioavailability [23, 24].

Extensive maternal cardiovascular adaptation takes place in order to sustain the development of the fetus. Blood volume and cardiac output increase, and there is a redistribution of blood flow to the different organs [25–28].

Distribution of polar drugs is limited mostly to the extracellular fluids, as the low lipid solubility impairs their diffusion through biological membranes and precludes their entrance into the intracellular space. During pregnancy, total body water increases as a result of intravascular and extravascular expansion, leading to modifications in the volume of distribution of the polar drugs [25]. Pregnancy modifies plasma proteins profile. Acute phase proteins, which include C-reactive protein, serum amyloid A, fibrinogen, and ceruloplasmine, are significantly increased [29, 30] whereas the increase in plasma volume results in a dilutional hypoalbuminaemia, as the synthesis of this protein is not modified. Changes in the concentration of plasma proteins, particularly albumin, may affect drug protein binding, modifying the free fraction of drugs. This fraction is the one that distributes extravascularly and reaches the site of action, thus is the pharmacologically active drug. If maternal albumin serum concentration is decreased, correspondingly, free fraction of drug may be increased. For highly bound drugs, such as AINEs, furosemide and digoxin, this may have clinical implications. However, if protein binding of the drug is maximal even at the low albumin concentrations that may be found during pregnancy, no pharmacokinetic modifications may be expected [31].

Opposite and unpredictable modifications due to pregnancy changes may be observed in the clearance of lipid-soluble drugs that are primarily eliminated by metabolism. Pregnancy may enhance drugs biotransformation by two mechanisms: increasing the access of the drug to the site of metabolism, particularly the liver and increasing the activity of the enzymatic system, particularly the hepatic cytochrome P-450 (CYP) family. Decreased binding to plasma proteins due to the pregnancy-related hypoalbuminaemia may increase liver metabolization of low hepatic extraction drugs, while increased hepatic blood flow may increase liver biotransformation of high hepatic extraction drugs. The activity of some drug metabolizing enzymes may be affected, either enhanced or decreased, by the action of the sexual steroids progesterone and oestradiol. During pregnancy, the expression of genes encoding hepatic CYP450 superfamily of microsomal enzymes may be decreased [16, 17, 32].

Increases in plasma volume and cardiac output and consequent redistribution of blood flow to the kidney produce marked increases in the glomerular filtration rate [26]. In consequence, plasma concentrations of hydrophilic, polar drugs may be lower, and half-life may be shorter when water-soluble drugs are given during pregnancy. 

Different pharmacokinetic behavior of antimicrobial agents in pregnant females of different species has been described [33–43]. For betalactams, kinetics may be greatly influenced by changes in the extracellular fluid and glomerular filtration rate. Increased volume of distribution and clearance leading to a decrease in the maternal serum concentrations has been described for piperacillin [33] and imipenem [34] in pregnant women and penicillin [35] in ewes. Cefatrizine's oral bioavailability was decreased in women during pregnancy [36]. In contrast, no changes related to pregnancy were found in the pharmacokinetics of amoxicillin [37], ceftriaxone [38], and the aminoglycoside tobramycin [39] in pregnant women. Opposite results were described for gentamicin, as its pharmacokinetic disposition did not change in mares [40] whereas increased clearance and decreased half-life was found in pregnant women [41, 42] and in ewes at the end of pregnancy [43].

## 3. Placental Functions and Drug Transfer

Several physiological and anatomical characteristics of the placenta (i.e., number of cellular layers between maternal and fetal circulation, endocrine activity, blood flow patterns, permeability to xenobiotics, and metabolizing activity) change during the pregnancy, and between species, resulting in a large variation in the placental function [7–9]. Differences in the thickness and surface area of the placenta, as well as differences in maternal and fetal blood flows, may explain some of the variations on the function of placenta between species, and may also explain increases in placental transfer during the late gestation [7]. In addition to dealing with endogenous compounds, the placenta metabolizes and transfers a large variety of pharmacological agents [44, 45]. Drugs may cross the placenta and reach the fetus by paracellular or transcellular mechanisms. To date, only transcellular diffusion and active transport have been identified as the major mechanisms involved in drug placental transfer. Type of passage is closely related to the physicochemical properties of the drug, as lipid solubility, pKa and degree of ionization, and molecular weight. Lipid-soluble, non ionized, small molecules with low protein binding can cross rapidly the placenta by diffusion depending only on the concentration gradient between maternal and fetal circulation and blood flow. Extensive plasma protein binding may interfere with the transfer of small, lipid-soluble drugs [31]. Water-soluble, highly ionized drugs are incapable to dissolve in the lipoprotein structure of the biological membranes. Thus, other mechanisms for reaching the fetal compartment, primarily carrier-related transport, play a major role. Placenta contains water-filled channels which may serve for the crossing of water-soluble substances. These pores are of different sizes between species; pore radius has been estimated to be 0.5 nm in sheep, and 15–30 nm in rabbits [7]. However, numerous tight junctions between cells impair this transplacental passage. More importantly, several carriers are differentially distributed between both maternal and fetal sides of the placenta for providing nutrients and disposing of waste products. These carriers may transfer xenobiotics with mechanisms similar to those used for their physiological function [45–50]. Transporters activity may have broad, overlapping substrate affinities. Some of these transporters prevent the transfer of xenobiotics from the maternal to the fetal compartment, primarily the P-glycoprotein, acting as a very effective efflux pump that extrudes substrates that were able to cross the cellular membrane to the extracellular fluid [45–49]. Other transporters identified in the placenta, that also perform as efflux pumps, are the breast cancer resistant protein (BCRP) and the multidrug resistance associated proteins (MRPs) [45, 46, 50]. Placental transporters may be effective limiting the passage of xenobiotics into the fetus. Moreover, one substance may be removed by more than one transporter, as it happens with glyburide, an oral hypoglycaemic drug, which is removed from the fetal compartment by BCRP and MRP3, both present in the maternal side of placenta [51–53]. On the contrary, another oral hypoglycaemic agent, metformin, which is a substrate for OTC1 and OTC2, present in liver and renal tissues, is not removed from placenta, as only OTC3 is present in this organ [54, 55]. The result is that metformin, but not glyburide, is detected in fetal tissues when diabetic women are treated with oral hypoglycaemic pharmacotherapy during pregnancy. On the contrary, other transporters located on the maternal and fetal side of the placenta [56–58] may be involved in the placental transfer of xenobiotics into the fetus. 

The placenta is able to metabolize xenobiotics since early pregnancy as this organ expresses various functionally active drug metabolizing enzymes [12, 14, 16, 17, 59–65]. Phase I metabolizing enzymes, such as CYP1A1, CYP4B1, and CYP19 (steroid aromatase, responsible of the placental conversion of androgens into estrogens) contribute to the oxidation of some xenobiotics in placenta [60, 61]. Several phase II enzymes, including glutathione transferases, N-acetyltransferase, sulfotransferase, and UDP-glucuronosyl transferase have been determined in the placenta [59]. However, placental ability to metabolize xenobiotics is less efficient than liver ability and changes with pregnancy stage. 

The concept of “placental barrier”, taken as an anatomical property of the placenta, is mistaken. The various functions of transport and biotransformation provided by the activity of carriers and metabolizing enzymes are the major components of the barrier effect that protects the fetus from the exposure of the pharmacological treatments applied to the mother. Thus, as with many biological functions, this protection may be successful for certain drugs and useless for others, and the safe view to take when using drugs during pregnancy is that drugs are always transferred to the fetus.

## 4. Antibiotic Therapy and Teratogenic Risk during Canine and Feline Pregnancy

To the authors' knowledge, there are few studies investigating the use of pharmacological agents during the canine and feline pregnancy. Most data in the first part of this paper were obtained from reports about human or animal (rhesus monkeys, sheep, mice, and rats) models of placental morphologies and functions, and similar experiences carried out in dogs and cats are scarce. As we have already stated, due to the relevant structural and functional diversity of the placenta among different species, data obtained in studies conducted in humans cannot be transferred to the pregnant dog or cat. However, the following data may be used to help in the decision of selecting a rational antimicrobial therapy during pregnancy in dogs and cats.

Physiological changes reported in the pregnant bitch resemble those described in pregnant women and consist of plasma volume expansion and increased blood volume, increased heart rate and cardiac output, increased gastric emptying time and decreased gastric and intestinal motility, and increased plasma renal flow and glomerular filtration rate [66–69]. Total plasma protein levels remain decreased or unchanged; acute phase proteins, including C-reactive protein and fibrinogen, increase in pregnancy, although it cannot be used as pregnancy diagnosis [29, 30]. Similar changes may occur in the queen. These modifications may not need dose adjustment; however, to our knowledge, no pharmacokinetic studies have been conducted comparing disposition of antimicrobials between pregnant and nonpregnant dogs or cats. 

Two major determinants of the extent of the potential effects of pharmacotherapy on the fetus are the drug itself (fetal concentrations and potential toxicity) and the stage of the pregnancy during the exposure. The fetus is at greater risk for teratogenic effects due to drug exposure during the first trimester of gestation. Critical time for embryotoxicity in the bitch is from 6 to 20 days after the preovulatory LH surge. At this time, the uterine fluid that bathes the embryo attains drug concentrations similar to those of the maternal circulation. Feline implantation occurs 12 to 13 days after ovulation [66–68]. Placentation in the cat and in the dog is endotheliochorial; that is, maternal and fetal circulations are separated by four tissue layers [67, 68]. To our knowledge, no studies have been conducted on the presence or absence of placental metabolizing enzymes or transporters in canine or feline placenta, and, as described above, these are major determinants of its barrier function. Therefore, it is very difficult to estimate with accuracy the ability of a drug to cross the canine or feline placenta, and thus, the exact risk of harming the fetus. 

A deletion mutation in the MDR1 gene has been identified as the cause of a functional P-glycoprotein defect in some canine breeds [70–72]. If P-glycoprotein is present in canine placenta, and its activity is as relevant as in human and rodent placenta, this genetic trait could be a source of differences in placental transfer of P-glycoprotein-substrate drugs. Antibiotics that may pose a threat to the fetus, as fluoroquinolones, including veterinary enrofloxacin, have been proved substrates for P-glycoprotein and BCRP [73–75].

Once drugs cross the placenta and enter the canine or feline fetal circulation via the umbilical vein, either may reach the liver (40%–80%) or bypass the liver via the ductus venosus which connects the umbilical vein with the caudal vena cava [68]. This shift allows maternal blood from the umbilical vein to deliver nutrient rich blood to the rest of the fetus. Fetal liver may uptake some xenobiotics and detoxify them, thus protecting the fetus from exposure of high levels of drugs. However, the enzymatic activity of the animal fetal liver is generally less than in adults [66].

Teratogenic risk of frequently used veterinary drugs in treating dogs and cats has been classified by Papich and Davis [66]. Antimicrobial agents that have been shown to be safe for use during pregnancy include betalactam antibiotics (penicillin G, ampicillin, amoxicillin, amoxicillin-clavulanic, carbenicillin, ticarcillin, and cephalosporins), macrolides, and lincosamides (clindamycin, erythromycin, and lincomycin). Betalactams are the first choice for treating infections during pregnancy because of their low risk of harming the fetus, and low transplacental passage, due mostly to simple diffusion [66, 67, 76–80]. Macrolides are usually used clinically for patients with allergy to betalactams. The placenta seems to produce an effective barrier reducing the fetal exposure when macrolide antibiotics are used to treat maternal infections, so these drugs are considered of low risk in fetal exposures. A prospective controlled multicenter study of clarithromycin in pregnant women found that the drug did not increase the rate of major malformations [81].

Nitrofurantoin, streptomycin, gentamicin, amikacin, tetracyclines (doxycycline, oxytetracycline), sulphonamides, trimethoprim, and metronidazole are contraindicated during pregnancy, because they have been shown to cause congenital malformations or embryotoxicity. However, since Papich's report, human clinical experience and recent literature have determined the existence of drugs that although having potential risks, they could be used cautiously, as some aminoglycosides (gentamicin, amikacin, and kanamicin) and fluoroquinolones. 

Aminoglycosides are polar drugs, highly ionized in the maternal plasma; however, they were found to cross the placenta, Human data suggest low risk to the fetus, but streptomycin is contraindicated due to proved toxicity to the fetal ear [77]. Gentamicin is currently used associated to ampicillin in human obstetrics for prophylaxis of neonatal infections related to group B streptococci [82]. Toxic effects on rat fetal kidneys have been determined at high doses [83] and in kidneys of children [84].

Fluoroquinolones are not drugs of first choice in pregnant women [76, 77, 79]; they are reserved to betalactam- or macrolide-intolerant patients. Fluoroquinolones cross the placenta to a low extent [85–88]. This restricted passage may be due to a decreased passive diffusion and/or an active extrusion from placenta by transporters. Fluoroquinolones are substrates for some placental transporters [73–75]. This fact may be the source of interspecies variability on the fetal exposure to fluoroquinolones and represents an incognita in canine pregnancy; as to our knowledge, no studies have been reported on the presence and activity of transporters in canine placenta, and breed differences due to different genotypes may exist [70–72]. Newer fluoroquinolones may cross the placenta in higher extent [89].

Concerns regarding the use of fluoroquinolones during pregnancy are related to some of their adverse effects, primarily to the production of arthropathies in young animals. The puppies are especially sensitive to developing cartilage lesions due to fluoroquinolones [90–92]. The toxicological profile of fluoroquinolones got more complex with the increasing number of compounds and frequent clinical use, and adverse effects as convulsions [93], photophobia [94], and ocular toxicity related to lens (in dogs) [95] and retina (in cats) [96, 97] have been described. These adverse effects are mostly seen after prolonged administration or high doses and after preclinical safety evaluations [96, 98]. A multicenter prospective controlled study in pregnant women exposed to quinolones failed to prove differences in the rate of malformations between their offspring and children of mothers not exposed (controls) [99].

## 5. Conclusions

Pharmacotherapy during pregnancy in all species may affect adversely the developing fetus; therefore, it should be avoided when possible. However, some clinical conditions that may represent a serious risk for the mother's health, and thus also to the fetus, must be treated, and bacterial infections are one of them. 

Changes on the disposition of antimicrobial agents during pregnancy raise a major concern. Not only the outcome of therapy may be impaired, as successful antimicrobial treatment depends on appropriate plasma and tissue concentrations, but also selection of resistant bacteria may occur if the antimicrobial concentrations are not high enough to provide full antibacterial activity [100, 101]. 

When deciding on an antimicrobial therapy in the pregnant bitch or queen, the pharmacotherapy must meet two criteria: it should be the best treatment for the mother, and it should represent the less risk for the fetus.

The best treatment to the mother means to choose an appropriate drug, with the maximal therapeutic efficacy and less adverse effects, and to administer it in an appropriate dosage. As in the small animal practice dosages are usually calculated in mg/kg body weight, there is less risk of subtherapeutic dosage than in human medicine, in which fixed adult doses are used, even in pregnant women. This is relevant in antimicrobial therapy to attain clinical success and minimize bacterial resistance.

The risk to the fetus is a result of the ability of a drug to reach the fetal circulation and to produce toxic effects to the fetus. Special care must be taken during the organogenesis period, that is, up to day 20 of pregnancy in dogs and cats. The potential of a drug to cross the placenta may be estimated based on the physicochemical properties of the drug (lipid solubility, molecular weight, degree of ionization, and protein binding), maternal concentrations (dose and route of administration, length of treatment) and functional capability of the placenta in a particular gestational period. This last issue is the most difficult to infer, as data on canine and feline placental blood flow type, and presence and activity of placental transporters and enzymatic systems are lacking. Therefore, the clinician must rely on the knowledge of the pharmacological properties of the drugs to infer the extent of the placental passage and risk to fetus and on data obtained in other species. Betalactams are the first choice drugs for treating infectious conditions in pregnant bitches and queens, and the wide variety of frequent pathogens included in their activity spectrum indicates them in many veterinary infections. Macrolides are also considered of low risk during pregnancy whereas further studies should be conducted with fluoroquinolones administered in pregnant dogs and cats.
